# MALDI-TOF MS for rapid detection and differentiation between Tet(X)-producers and non-Tet(X)-producing tetracycline-resistant Gram-negative bacteria

**DOI:** 10.1080/21505594.2021.2018768

**Published:** 2021-12-24

**Authors:** Zi-Jian Zheng, Ze-Hua Cui, Qiu-Yue Diao, Xin-Qing Ye, Zi-Xing Zhong, Tian Tang, Shuai-Bin Wu, Hui-Ling He, Xin-Lei Lian, Liang-Xing Fang, Xi-Ran Wang, Li-Jie Liang, Ya-Hong Liu, Xiao-Ping Liao, Jian Sun

**Affiliations:** a National Risk Assessment Laboratory for Antimicrobial Resistance of Animal Original Bacteria, South China Agricultural University, Guangzhou, China; b Guangdong Laboratory for Lingnan Modern Agriculture, Guangzhou, China; c Guangdong Provincial Key Laboratory of Veterinary Pharmaceutics Development and Safety Evaluation, South China Agricultural University, Guangzhou, China; d Jiangsu Co-Innovation Center for the Prevention and Control of Important Animal Infectious Disease and Zoonosis, Yangzhou University, Yangzhou, China

**Keywords:** Rapid detection, phenotypic detection, MALDI-TOF MS, tetracycline resistance, Tet(X)-producers, non-Tet(X)-producers

## Abstract

The extensive use of tetracycline antibiotics has led to the widespread presence of tetracycline-resistance genes in Gram-negative bacteria and this poses serious threats to human and animal health. In our previous study, we reported a method for rapid detection of Tet(X)-producers using MALDI-TOF MS. However, there have been multiple machineries involved in tetracycline resistance including efflux pump, and ribosomal protection protein. Our previous demonstrated the limitation in probing the non-Tet(X)-producing tetracycline-resistant strains. In this regard, we further developed a MALDI-TOF MS method to detect and differentiate Tet(X)-producers and non-Tet(X)-producing tetracycline-resistant strains. Test strains were incubated with tigecycline and oxytetracycline in separate tubes for 3 h and then analyzed spectral peaks of tigecycline, oxytetracycline, and their metabolite. Strains were distinguished using MS ratio for [metabolite/(metabolite+ tigecycline or oxytetracycline)]. Four control strains and 319 test strains were analyzed and the sensitivity was 98.90% and specificity was 98.34%. This was consistent with the results obtained from LC-MS/MS analysis. Interestingly, we also found that the reactive oxygen species (ROS) produced by tetracycline-susceptible strains were able to promote the degradation of oxytetracycline. Overall, the MALDI^Tet(X)-plus^ test represents a rapid and reliable method to detect Tet(X)-producers, non-Tet(X)-producing tetracycline-resistant strains, and tetracycline-susceptible strains.

## Introduction

Tetracyclines are a group of broad-spectrum antibiotics used extensively for more than 70 years for the prophylaxis and treatment of human and animal bacterial infections [1]. The tetracyclines inhibit protein synthesis by preventing the attachment of aminoacyl-tRNAs to the ribosomal acceptor (A) site [2]. However, the extensive use of tetracycline antibiotics in medicine and agriculture has resulted in the maintenances of a continuous selective pressure for tetracycline resistance in previously susceptible bacteria [3]. High levels of the drug are present in surface water and groundwater as well as sludge and sediment in waste treatment plants. Tetracycline-resistant bacteria have even been isolated from cooked meat products, waterfowl, and ready-to-eat foods [4–8]. The pervasive presence of tetracycline-resistance genes and bacteria poses serious threats to human and animal health.

Tetracycline resistance is primarily the result of efflux and ribosome protection as well as enzymatic inactivation that serves to lower intracellular concentrations of tetracyclines and protect the bacterial ribosome from tetracycline binding [1,9,10]. The current methods used for the detection of tetracycline-resistant bacteria include PCR and whole-genome sequencing (WGS) that are used to correlate resistance genotypes with phenotypes [11,12]. The latter is most often the calculation of the minimal inhibitory concentration (MIC) of the drug [13]. The disadvantage of these methods is that they are time-consuming. Therefore, a rapid and reliable detection method for tetracycline-resistant Gram-negative bacteria would assist clinical treatment regimens.

In our previous study, we developed the tetracycline inactivation method (TIM) and the MALDI^Tet(X)^ test method that can rapidly detect high-level TGC resistance in Tet(X)-producing Gram-negative bacteria [14,15]. To our knowledge, there is currently no detection method for different tetracycline-resistant Gram-negative bacteria using matrix-assisted laser desorption ionization-time of flight mass spectrometry (MALDI-TOF MS). Here, in the present work, reported MALDI^Tet(X)-plus^, a MALDI-TOF MS-based method to probe and distinguish the tetracycline-resistant and susceptible strains from non-Tet(X) producing strains. The established method provided a highly reliable and rapid approach to comprehensively profile the tetracycline-resistant strains among bacterial population in a high-throughput manner. Interestingly, the tetracycline-susceptible strains were also observed to promote the degradation of tetracycline antibiotic. This could be explained that tetracycline degraded in response to the ROS generated by the bacteria during the antibiotic stress.

Overall, our work was able to identify the Tet(X)-producers, non-Tet(X) producers and differentiate the phenotypically resistant and susceptible strains from non-Tet(X) producers, which showed great potential to profile tetracycline-resistant bacteria for further application [16].

## Materials and methods

### Bacterial strains

The strains used in the current study were isolated between June 2016 and Nov 2018 as previously described, and have been sequenced and stored in our archived collection [15,17]. The 319 isolates were previously confirmed to have *tet* genes that included those for efflux pumps [*tet*(A), *tet*(B), *tet*(D), *tet*(G)], ribosomal protection [*tet*(M)], and enzymatic modification [*tet*(X3), *tet*(X4), *tet*(X2)-*tet*(X6), *tet*(X3)-*tet*(X6)]. Within this group there were 124 Tet(X)-producers including 38 *tet*(X3), 1 *tet*(X2)-*tet*(X6) and 16 *tet*(X3)-*tet*(X6) positive *Acinetobacter* spp. as well as 69 *tet*(X4) positive *Escherichia coli* strains. The remaining 195 non-Tet(X)-producers included 149 tetracycline-resistant and 46 tetracycline-susceptible strains encompassing 16 *Acinetobacter* spp.[2 *tet*(A), 12 *tet*(B), 1 *tet*(M), 1 *tet*(A)-*tet*(B)-*tet*(D)], 102 *E. coli* [49 *tet*(A), 16 *tet*(B), 3 *tet*(D), 1 *tet*(G), 5 *tet*(M), 5 *tet*(A)-*tet*(B), 13 *tet*(A)-*tet*(M), 2 *tet*(B)-*tet*(D), 1 *tet*(B)-*tet*(M), 6 *tet*(D)-*tet*(M), 1 TMexCD1-TOprJ1], 15* K. pneumoniae* [9 *tet*(A), 1 *tet*(B), 1 *tet*(D), 1 *tet*(A)- *tet*(M), 3 *tet*(A)- *tet*(D)], 16 *Salmonella enteritis* [12 *tet*(B), 2 *tet*(A)- *tet*(B), 1 *tet*(B)- *tet*(D), 1 *tet*(B)- *tet*(M)]. The isolates that lacked *tet* genes included 39 *E. coli* and 7 *K. pneumoniae* strains. Four control strains and 90 non-Tet(X)-producers test strains were used to establish the MALDI^Tet(X)-plus^ test (Table 1) and 229 test strains including 124 Tet(X)-Producers and 105 non-Tet(X)-producers were used to test its sensitivity and specificity (Table 2).

**Table 1.** Bacterial strain characteristics and corresponding the MALDI^Tet(X)-plus^ test results

			MIC						MS Ratio	
Species	n	genes	TC(1s)^a^	OTC(1s)^a^	DOX(2s)^a^	TGC(3s)^a^	ERA(4s)^a^	OMA(4s)^a^	TGC	OTC
Control strains	4									
*E. coli-*JM109-pBAD24-tet(X3)	1	*tet*(X3)	64	128	32	4	4	16	0.5 ± 0.09	0.2283 ± 0.0503
*E. coli-*JM109-pBAD24-tet(X4)	1	*tet*(X4)	64	128	16	8	2	16	0.43 ± 0.11	0.2155 ± 0.013
*E. coli* JM109 – pBAD24	1	-	2	2	0.5	0.03	0.008	0.125	0 ± 0	0.1346 ± 0.0629
*E. coli* 25,922	1	-	2	2	0.5	0.03	0.06	0.25	0 ± 0	0.1221 ± 0.0498
Test strains	90									
Non-Tet(X)-producers in tetracycline-resistant strains	61									
*Acinetobacter* spp.	2	*tet*(A)	>256	>256	128–256	2–4	1–2	8	0 ± 0	0.0041 ± 0.0058 to 0.0151 ± 0.0132
*Acinetobacter* spp.	6	*tet*(B)	>256	>256	128–256	0.5–4	0.5–1	0.5–8	0 ± 0	0 ± 0 to 0.0463 ± 0.0208
*E. coli*	17	tet(A)	64->256	64->256	4->256	0.06–4	0.03–2	0.25–8	0 ± 0	0 ± 0 to 0.0189 ± 0.0157
*E. coli*	7	tet(B)	128->256	128->256	32->256	0.125–2	0.03–1	0.5–8	0 ± 0	0 ± 0 to 0.0158 ± 0.0224
*E. coli*	1	tet(G)	256	256	64	2	0.5	8	0 ± 0	0 ± 0
*E. coli*	4	tet(M)	128–256	128->256	32–64	0.125–0.5	0.125–0.25	2	0 ± 0	0 ± 0
*E. coli*	4	*tet*(A)- *tet*(B)	128->256	>256	64–256	0.03–0.5	0.03–0.25	0.25–4	0 ± 0 to 0.0035 ± 0.005	0 ± 0 to 0.0111 ± 0.0157
*E. coli*	4	*tet*(A)- *tet*(M)	64–256	64->256	32->128	0.25–0.5	0.125–0.25	2–4	0 ± 0 to 0.0085 ± 0.0121	0 ± 0 to 0.0727 ± 0.0748
*K. pneumoniae*	3	*tet*(A)	128->256	256->256	32–256	0.5–4	0.125–2	4–8	0 ± 0 to 0.0033 ± 0.0047	0 ± 0 to 0.0518 ± 0.0309
*K. pneumoniae*	1	*tet*(D)	256	>256	128	2	1	8	0 ± 0	0.0231 ± 0.0326
*K. pneumoniae*	3	*tet*(A)- *tet*(D)	>256	>256	>256	1–2	1	8	0 ± 0	0.0128 ± 0.0137 to 0.0419 ± 0.0326
*S. enteritis*	5	*tet*(B)	64–256	128->256	8–64	0.5–2	0.06–0.5	1–4	0 ± 0	0 ± 0
*S. enteritis*	2	*tet*(A)- *tet*(B)	128	>256	64	1	0.25	2	0.0003 ± 0.0004 to 0.0003 ± 0.0005	0 ± 0 to 0.0083 ± 0.0117
*S. enteritis*	1	*tet*(B)- *tet*(D)	256	128	64	1	0.25	4	0 ± 0	0 ± 0
*S. enteritis*	1	*tet*(B)- *tet*(M)	256	>256	64	1	0.125	2	0 ± 0	0.0136 ± 0.0193
Tetracycline-susceptible strains	29									
*E. coli*	29	non-*tet*(X)^b^	0.5–1	1–2	1	0.125–0.5	0.03–0.06	0.25–1	0 ± 0 to 0.0018 ± 0.0025	0.0671 ± 0.0227 to 0.3897 ± 0.0184

TC, tetracycline; OTC oxytetracycline; DOX, doxycycline; TGC, tigecycline; ERA, eravacycline; OMA, omadacycline.

^a^The number in parentheses indicates the generation of tetracycline.

^b^Non-*tet*(X) strains lack all *tet* genes as well as *tet*(X).

**Table 2.** Characteristics of test strains used for test validation

			MIC						MS Ratio	
Species	n	genes	TC(1s)^a^	OTC(1s)^a^	DOX(2s)^a^	TGC(3s)^a^	ERA(4s)^a^	OMA(4s)^a^	TGC	OTC
Test strains	229									
Tet(X)-producers	124									
*Acinetobacter* spp.	38	*tet*(X3)	64->256	16->256	1–128	16–64	4–32	4–64	0.0134 ± 0.0038 to 0.5778 ± 0.1437	0.0316 ± 0.0089 to 0.2793 ± 0.1123
*Acinetobacter* spp.	1	*tet*(X2)-*tet*(X6)	>256	256	128	32	4	16	0.231 ± 0.1276	0.2656 ± 0.0449
*Acinetobacter* spp.	16	*tet*(X3)-*tet*(X6)	32->256	8->256	8–128	16–64	4–16	4–64	0.0117 ± 0.0165 to 0.3845 ± 0.1291	0.0415 ± 0.0257 to 0.1987 ± 0.0493
*E. coli*	69	*tet*(X4)	32->256	32->256	32–128	1–32	1–16	8–64	0.0008 ± 0.0011 to 0.4812 ± 0.0918	0.0104 ± 0.0089 to 0.2097 ± 0.0298
Non-Tet(X)-producing tetracycline-resistant strains	88									
*Acinetobacter* spp.	6	*tet*(B)	>256	>256	64–256	2–4	0.25–1	2–8	0 ± 0	0 ± 0 to 0.0271 ± 0.0192
*Acinetobacter* spp.	1	*tet*(M)	>256	>256	128	2	1	4	0 ± 0	0.0094 ± 0.0133
*Acinetobacter* spp.	1	*tet*(A)- *tet*(B)-*tet*(D)	>256	>256	64	2	1	4	0 ± 0	0 ± 0
*E. coli*	32	tet(A)	64–256	64->256	16–256	0.125–2	0.06–2	0.125–8	0 ± 0	0 ± 0 to 0.0344 ± 0.0255
*E. coli*	9	tet(B)	256->256	256->256	64->256	0.25–1	0.06–0.5	0.25–8	0 ± 0	0 ± 0 to 0.0704 ± 0.0629
*E. coli*	3	tet(D)	256	64->256	32–128	0.25–4	0.25–2	4–8	0 ± 0	0 ± 0
*E. coli*	1	tet(M)	128	256	128	0.25	0.125	2	0 ± 0	0 ± 0
*E. coli*	1	*tet*(A)- *tet*(B)	64	64	16	0.125	0.25	2	0 ± 0	0 ± 0
*E. coli*	9	*tet*(A)- *tet*(M)	256->256	64->256	32->256	0.25–2	0.06–1	1–4	0 ± 0 to 0.0147 ± 0.0208	0 ± 0 to 0.0287 ± 0.0113
*E. coli*	2	*tet*(B)- *tet*(D)	256->256	>256	32->256	0.25–4	0.25–2	4–16	0 ± 0 to 0.0005 ± 0.0008	0 ± 0 to 0.0237 ± 0.0241
*E. coli*	1	*tet*(B)- *tet*(M)	>256	>256	256	0.5	0.125	1	0 ± 0	0.0235 ± 0.0332
*E. coli*	6	*tet*(D)- *tet*(M)	128->256	>256	32->256	0.25–4	0.25–3	2–8	0 ± 0	0 ± 0 to 0.0939 ± 0.0332
*E. coli*	1	TMexCD1- TOprJ1	16	16	32	4	8	16	0 ± 0	0.0581 ± 0.0170
*K. pneumoniae*	6	*tet*(A)	256->256	>256	64	0.25–0.5	0. 25–0.5	2–4	0 ± 0 to 0.0168 ± 0.0237	0 ± 0 to 0.017 ± 0.024
*K. pneumoniae*	1	*tet*(B)	128	256	256	0.5	0.125	1	0 ± 0	0 ± 0
*K. pneumoniae*	1	*tet*(A)- *tet*(M)	128	128	32	0.5	0.25	2	0 ± 0	0 ± 0
*S. enteritis*	7	*tet*(B)	128	128->256	32–64	0.5	0.06–0.125	2–4	0 ± 0	0 ± 0 to 0.006 ± 0.0085
Tetracycline-susceptible strains	17									
*E. coli*	10	non-*tet*(X)^b^	0.5–1	2	1	0.125–0.25	0.03–0.06	0.5	0 ± 0 to 0.0004 ± 0.0005	0.0465 ± 0.0103 to 0.3929 ± 0.1155
*K. pneumoniae*	7	non-*tet*(X)^b^	4–8	4–8	2–4	0.5–1	0.25–0.5	1–4	0 ± 0	0.0619 ± 0.0104 to 0.2202 ± 0.0255

TC, tetracycline; OTC oxytetracycline; DOX, doxycycline; TGC, tigecycline; ERA, eravacycline; OMA, omadacycline.

^a^The number in parentheses indicates the generation of tetracycline.

^b^Non-*tet*(X) strains lack all *tet* genes as well as *tet*(X).

These test strains were isolated from feces (297), dust (3), sewage (8), flower (one), and soil (9) samples, and 1 *E. coli* (TMexCD1-TOprJ1) was provided by professor Jian-Hua Liu (South China Agricultural University). The fecal samples were collected from chickens, ducks, geese, pigs, and patients at a tertiary hospital in Guangdong **(Supplementary Table 1)**. All test strains were identified by MALDI-TOF MS (Axima-Assurance-Shimadzu).

### Antibiotic susceptibility testing

Antimicrobial susceptibility assay was performed and interpreted according to the CLSI guidelines [18]. Tigecycline (TGC C29H39N5O8, MW: 585.65, purity > 95%), oxytetracycline (OTC C22H24N2O9, MW: 496.89, purity > 95%), tetracycline (TC) and doxycycline (DOX) were purchased from Yuanye Biotechnology (Shanghai, China). Omadacycline (OMA) and eravacycline (ERA) were purchased from MedChemExpress (Monmouth Junction, NJ, USA). Antibiotic stocks solutions were prepared according to the manufacturer’s recommendations. All antibiotic solutions (5000 mg/L) were stored at −80°C for 2 months. The MICs of tetracycline, oxytetracycline, and doxycycline were determined using the agar dilution method and the microdilution broth method was used for tigecycline, omadacycline, and eravacycline. *E. coli* ATCC 25922 was used as the quality control strain. Susceptibilities of TGC for *Acinetobacter* spp. and *E. coli* strains were interpreted according to the FDA criteria (susceptible, ≤2 mg/L; intermediate, 4 mg/L; resistant, ≥8 mg/L). [14; 17]

### Detection of tetracycline resistance genes

Genomic DNA from the 319 test strains were examined using WGS and the reads were assembled using SPAdes v3.6.2 and the datasets can be found in previous study. [14, 19; 17] PCR was used to determine whether the tetracycline-resistance mechanism belonged to the efflux, ribosome protection, or enzymatic inactivation by screening for the presence of *tet*(X3), *tet*(X4), *tet*(X6), *tet*(A), *tet*(B), *tet*(D), *tet*(G) and *tet*(M) as previously described [15,17,20–22]. Antibiotic resistance genes (ARG) were analyzed using the CGE server (https://cge.cbs.dtu.dk/services/) and ABRicate (https://github.com/tseemann/abricate) [13].

### The MALDI^Tet(X)-plus^ test

**Figure 1.**
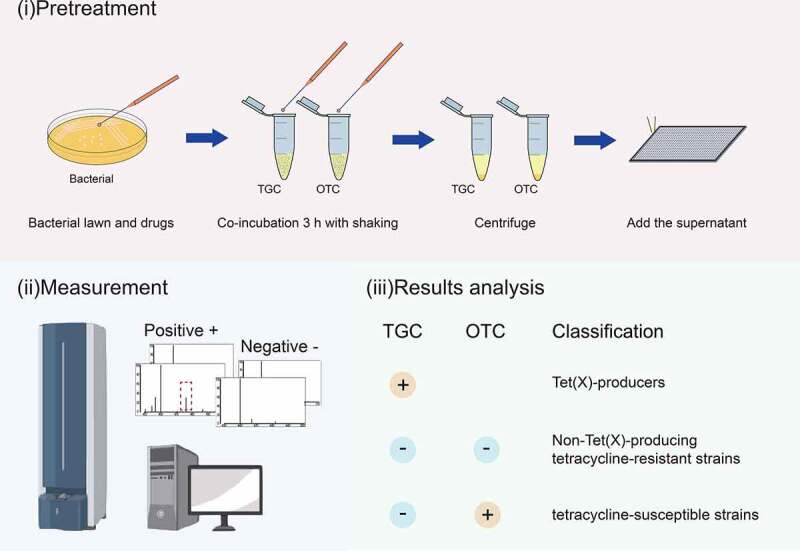
Strategy for identification of Tet(x)-producers and non-Tet(X)-producing tetracycline-resistant strains and tetracycline-susceptible strains using the MALDI^Tet(X)-plus^ test. Strains that showed peaks of tigecycline metabolite or oxytetracycline metabolite were labeled with “+”

TGC and OTC were used as substrates for the development of the MALDI^Tet(X)-plus^ test. A 10 μL loopful of overnight bacterial cultures were used to detect TGC and OTC degradation. The sample was added in 500 μL of 0.5% NaCl containing either 50 mg/L TGC or OTC in Eppendorf tubes and vortexed for 1 min and were incubated at 37°C with shaking in the dark for 3 h. These samples were centrifuged for 3 min at 14,000 × g and 1 μL of the clear supernatant was spotted onto an MSP 384 target polished steel plates (Shimadzu, Kyoto, Japan) and allowed to dry at room temperature for 3 min before 1 μL of the matrix (HCCA, α-cyano-4-hydroxycinnamic acid, C10H7NO3, MW: 189.17, purity > 99%, Sigma-Aldrich, St. Louis, MO, USA) was overlaid onto each target spot. The peak of the HCCA polymer (m/z 378 ± 0.2) was used as the internal calibration of the mass spectrometer. Mass spectra were acquired using a Shimadzu performance mass spectrometer and Shimadzu Biotech MALDI-MS software operating in positive linear ion mode between 100 and 1,000 Da. The parameters were set as follows: ion source 1, 20 kV; ion source 2, 2.62 kV; lens, 6 kV; pulsed ion extraction, 114 ns; electronic gain, enhanced; mode, low range; mass range selection, 50 to 1,050 Da; laser frequency, 60 Hz; digitizer trigger level, 2,500 mV; laser attenuator, 25%; and laser range, 40%. A total of 500 shots were acquired in each position for each spectrum (Figure 1).

### Spectral analysis

**Figure 2.**
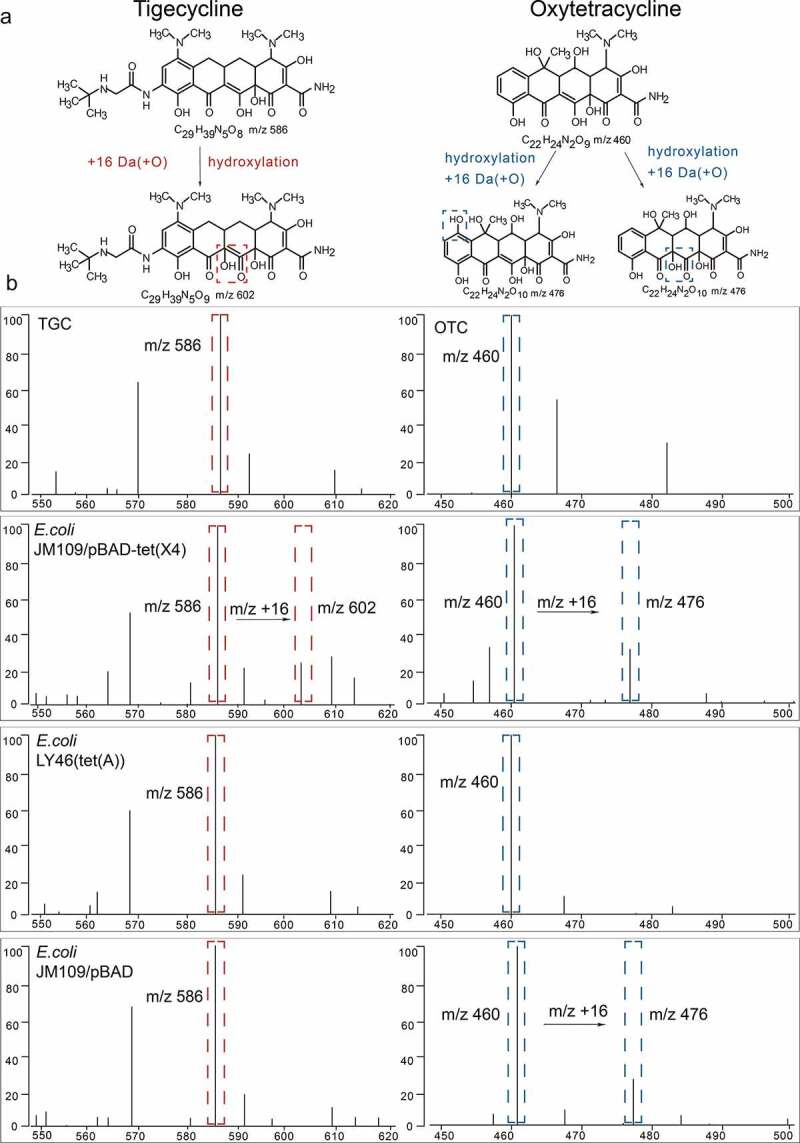
Representative MALDI-TOF MS results for detection of Tet(x)-producers and non-Tet(X)-producers. (a) Structures of TGC and OTC along with their oxygen-modified derivatives. (b) Representative MALDI-TOF MS spectra of tigecycline and oxytetracycline oxygenation assays after a 3 h incubation. Peaks of tigecycline and tigecycline metabolite are denoted by dashed red lines and represent peaks at m/z 586 ± 0.2 and m/z 602 ± 0.2, respectively. Peaks of oxytetracycline and oxytetracycline metabolite are denoted by dashed blue lines and represent peaks at m/z 460 ± 0.2 and m/z 476 ± 0.2, respectively

TGC peaks were analyzed and MS ratios [metabolite/(metabolite + TGC)] [M/(M + T)] were calculated as described for the MALDI^Tet(X)^ test [14]. Peaks for OTC (m/z 460 ± 0.2) and their metabolites (C_22_H_24_N_2_O_10_, m/z 476 ± 0.2) were manually labeled and their intensities were recorded. MS ratios of intensities were calculated as: [metabolite/(metabolite+OTC)] [M/(M + O)]. The threshold ratio was established using 90 non-Tet(X)-producers that included 61 tetracycline-resistant strains and 29 tetracycline-susceptible strains. All experiments were carried out on three independent bacterial cultures on three different days (Figure 2). The MS ratios [M/(M + T)] were used to detect Tet(X)-producing strains as described for the MALDI^Tet(X)^ test. The MALDI^Tet(X)-plus^ test method combines the MS ratios [M/(M + T)] and the MS ratios [M/(M + O)] to distinguish Tet(X)-Producers and Non-Tet(X)-Producing tetracycline-resistant Gram-negative bacteria.

### Statistical analysis

Descriptive analyses of MS ratios were performed using functions provided in Excel 2010 (Microsoft) and R software. The optimal cutoff value was performed by receiver operating characteristic (ROC) curve analysis with GraphPad Prism 9 (GraphPad San Diego, CA, USA) [23]. The optimal cutoff point was defined by the Youden index [24]. The ratio-based model was validated for the results of 229 well-characterized isolates that had been previously identified using PCR.

### LC–MS/MS analysis

A total of 15 strains with different resistance genes were selected to compare the ability to degrade OTC. Strains were incubated using the same method as the MALDI^Tet(X)-plus^ test. Then, μL of the supernatant was diluted to 5 mL using the mobile phase. After filtration with 0.22 μm filter, the concentrated extract was separated using a Waters symmetry C18 (100 mm × 2.1 mm, 3.5 μm, Agilent Technologies, Palo Alto, CA) in a liquid chromatograph equipped with a quaternary pump, an autosampler and a column oven (Agilent series 1200, Agilent, Santa Clara, USA) coupled to a triple quadrupole mass spectrometer (API 4000, AB SCIEX™). The mobile phase consisted of ACN (10%) and 0.01 M formic acid (90%) for OTC, Chromatographic separation was performed using a mobile phase consisting of 0.1% formic acid in water (Phase A) and 0.1% formic acid in methanol (Phase B) that formed the following gradient: 0–0.5 min, 5% B; 0.5–3.0 min, 5% to 80% B; 3.0–4.0 min, 80% B; 4.0–4.1 min, 80% to 5% B; 4.1–15.0, 5% B. The flow rate was 0.2 mL/min, the injection volume was 10 μL. The mass spectrometric analysis was performed in the positive electrospray ionization mode (ESI) and multiple-reaction monitoring (MRM) mode. The MRM transitions monitored were 586.4 to 569.3 for TGC and 461.2 to 426.2 for OTC The ion source-dependent parameters were as follows: temperature (TEM), 550°C; high collision gas; curtain gas(CUR), 40 Psi; ion source gas 1(GS1), 50 Psi; ion source gas 2(GS2), 50 Psi.

### Contribution of H_2_O_2_ to OTC metabolite

*E. coli* ATCC 25922 was exposed to low-level H_2_O_2_ (0.1 mM H_2_O_2_) for 30 min to improve the level of resistance against oxygen stress before treating OTC (50 mg/L). The H_2_O_2_ was removed by centrifugation at 4 000 x g for 10 min and strains were allowed to recover at 37°C for 90 min until all groups had the same OD_600_ of 0.2. And then, serial samples were obtained at 0, 1, 2, and 9 h after incubation with OTC at 37°C. Bacterial counts were determined based on the quantitative cultures on Lysogeny Broth (LB medium) plates. Non-treated *E. coli* ATCC 25922 were used as a control. Five independent experimental runs were performed. Results were performed using functions provided in Excel 2010 (Microsoft) and R software.

## Results

### Antibiotic susceptibility testing

The 319 strains used for test development possessed TGC and OTC MICs that ranged from 0.03 to 64 mg/L and 1 to >256 mg/L. Non-Tet(X)-producers including 58/61 were TGC susceptible and 3 intermediate. All non-Tet(X)-producing tetracycline-resistant strains were OTC resistant and all 29/29 tetracycline-susceptible strains were susceptible to both TGC and OTC (Table 1).

The validate strains included 122 strains that were TGC resistant and 2 single *E. coli* [*tet*(X4)] isolates that were TGC intermediate or susceptible. Non-Tet(X)-producing tetracycline-resistant strains included 80 were TGC susceptible strains and 7 TGC intermediate, and all were OTC resistant. Tetracycline-susceptible strains included 17 isolates that were all TGC susceptible, and 12 OTC susceptible, 5 OTC intermediate (Table 2).

### Detection of different tetracycline-resistant strains using the MALDI^Tet(X)-plus^ test

We established cutoff values for M/(M + T) as 0.00405 and non-Tet(X)-producers possessed values <0.00405 [14]. In the present study, the M/(M + O) of 61 non-Tet(X)-producing tetracycline-resistant strains ranged from 0 ± 0 to 0.0727 ± 0.0748, and 29 tetracycline-susceptible strains ranged from 0.0671 ± 0.0227 to 0.3897 ± 0.0184. ROC analysis for the M/(M + O) values allowed us to define a cutoff value at 0.05945 that discriminated tetracycline-resistant strains from tetracycline-susceptible strains. Non-Tet(X)-producers with MS ratios M/(M + O) <0.05945 were classified as tetracycline-resistant strains. All tetracycline-susceptible strains in the non-Tet(X)-producer group possessed had M/(M + O) values >0.05945. This indicated that the sensitivity MALDI^Tet(X)-plus^ test was 98.36% and the specificity was 100% **(Figure S1)**.

### Model Validation

**Figure 3.**
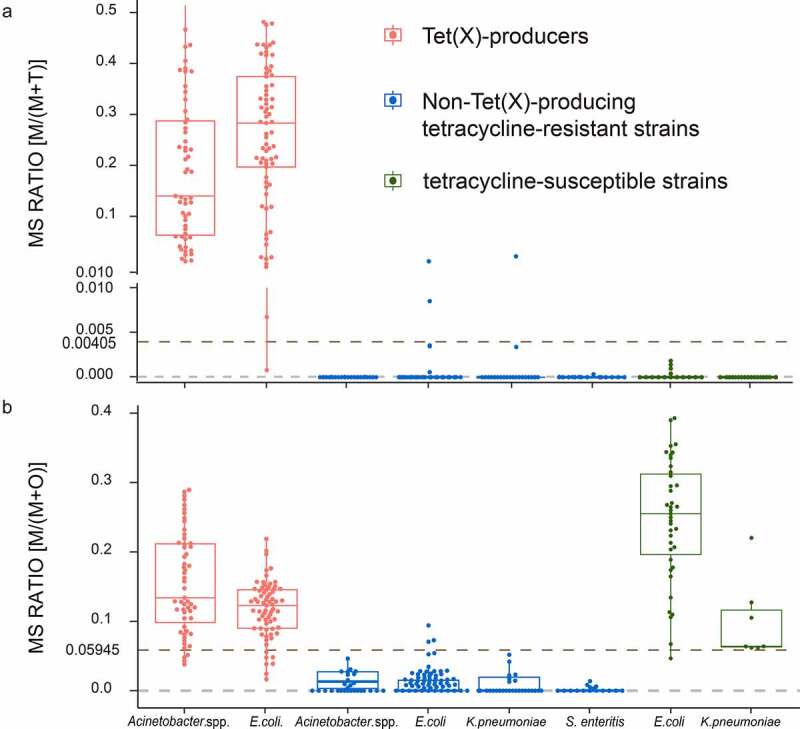
MALDI^Tet(x)-plus^ test results using 319 test strains. (a) The cutoff value of 0.00405 can clearly distinguish Tet(X)-producers M/(M + T) >0.00405 (b) The cutoff value of 0.05945 can clearly identify non-Tet(X)-producers possessing M/(M + O) <0.05945. Three independent experiments were performed

The MALDI^Tet(X)-plus^ test was validated using 229 strains including 124 Tet(X)-producers, and 88 non-Tet(X)-producing tetracycline-resistant and 17 tetracycline-susceptible strains. The MS ratio of M/(M + T) for the 124 Tet(X)-producers ranged from 0.0008 ± 0.0011 to 0.5778 ± 0.1437, and the non-Tet(X)-producers ranged from 0 ± 0 to 0.0168 ± 0.0237. The sensitivity using the validation group was 99.19% and specificity was 98.10% **(Figure S2)**. In addition, M/(M + O) values for all the Tet(X)-producers ranged from 0.0104 ± 0.0089 to 0.2793 ± 0.1123 (Figure 3(a)).

In the non-Tet(X)-producers group, the MS ratios of M/(M + O) of 88 tetracycline-resistant strains ranged from 0 ± 0 to 0.0704 ± 0.0629. The sensitivity using the validation group was 97.73% and specificity was 94.12%. Similarly,17 tetracycline-susceptible strains possessed M/(M + O) ranging from 0.0465 ± 0.0103 to 0.3929 ± 0.1155 **(Figure S3)**.

In conclusion, among 319 test strains, the MS ratios for M/(M + T) ranged from 0 ± 0 to 0.5778 ± 0.1437 and the MS ratios M/(M + O) ranged from 0 ± 0 to 0.3929 ± 0.1155. Seven test strains were misclassified using the MALDI^Tet(X)-plus^ test. The sensitivity was 98.90% and specificity was 98.34% (Figure 3(b)).

### LC–MS/MS analysis

**Figure 4.**
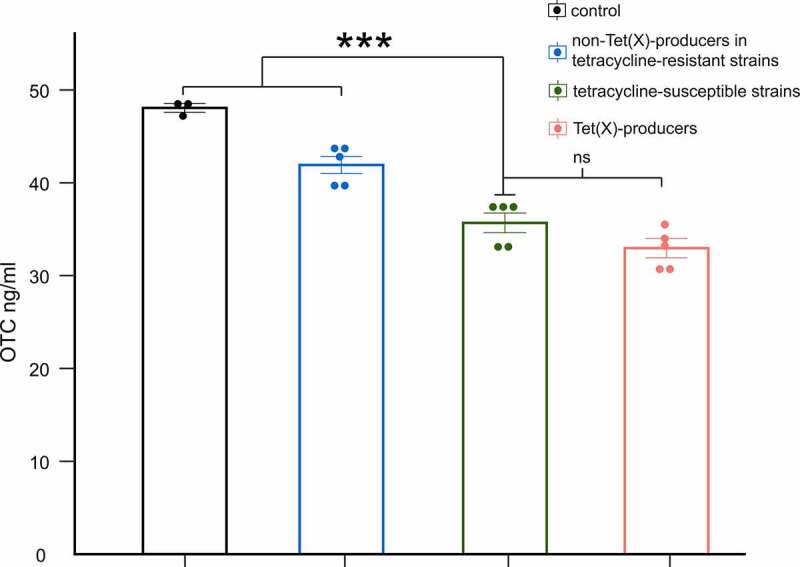
LC-MS/MS results for degradation of OTC by Tet(x)-producers, non-Tet(X) producers in tetracycline-resistant strains, and tetracycline-susceptible strains. The control group includes only OTC. We compare tetracycline-susceptible strains with Tet(X)-producers, non-Tet(X) producers in tetracycline-resistant strains, and the control group (three asterisk for *p* < 0.001)

The LC-MS/MS was used to assess the degradation of OTC after incubation with tested strains for 3 h. Respectively, the degradation efficiencies of OTC by Tet(X)-producing strains, non-Tet(X)-dependent tetracycline-resistant strains, and tetracycline-susceptible strains in 3 h ranged from 24.67% to 36.73%, 8.87% to 17.60%, and 21.74% to 31.76%. The degradation of OTC slightly increased (5.66%) after incubation with Tet(X)-producers, yet the difference was not significant (*p* = 0.1056). Interestingly, the degradation by tetracycline-susceptible strains was observed ~2-fold larger than that by non-Tet(X)-dependent tetracycline-resistant strains (*p* < 0.001) (Figure 4).

### Involvement of ROS to OTC degradation

**Figure5:**
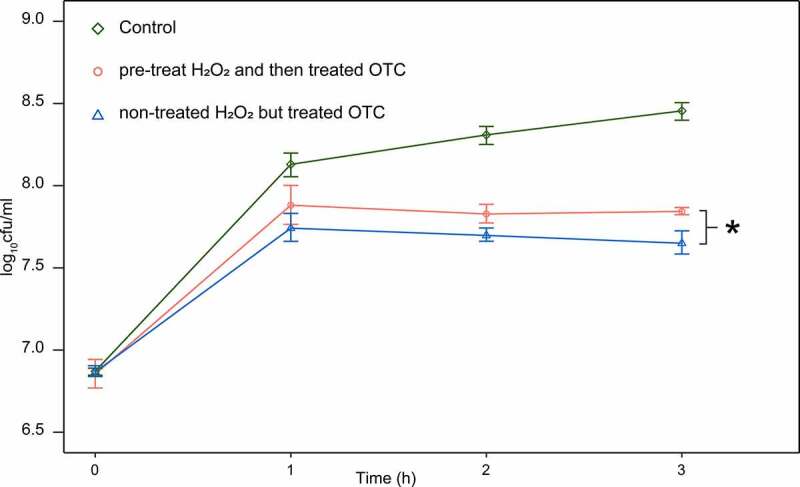
Strain survival from 50 mg/L OTC, after pre-treated H_2_O_2_ for 30 min and compared with non-pre-treated strain (one asterisk for *p* < 0.05). control group grown in LB medium without H_2_O_2_ and OTC. Curves represent the mean of five independent cultures per time point

To further investigate the potential mechanism of OTC-degradation by tetracycline-susceptible strains, we sought to determine whether the ROS generated during antibiotic exposure was involved in this unexpected degradation. The result indicated that after incubation with OTC for 3 h, H_2_O_2_ pre-treated group generally demonstrated better growth comparing with non-pre-treated group, by showing significantly higher viable cells (*p* < 0.05) (Figure 5).

## Discussion

MALDI-TOF MS has become an established tool for the identification of microbes and it can usually meet requirements for the analysis of biological samples because it enables molecular-weight determinations and is a rapid, relatively simple test with high sensitivity [25,26]. In the present study, based on MALDI-TOF MS, we develop the MALDI^Tet(X)-plus^ test to detect Tet(X)-producers, non-Tet(X)-producing tetracycline-resistant and tetracycline-susceptible Gram-negative bacteria. This is a high-throughput method that utilized polished steel plates containing 384 target sites and maintains excellent sensitivity and specificity.

Compared to the current methods for the detection of tetracycline-resistant bacteria, the MALDI^Tet(X)-plus^ test requires only three steps that can be completed in 3 h. Many genotypic and phenotypic methods are currently in use to distinguish between Tet(X)-producers and non-Tet(X)-producing tetracycline-resistant Gram-negative bacteria. For example, traditional PCR and multiplex PCR methods are genotypic detection methods with high sensitivity and specificity but these methods are unable to identify unknown genes [27,28]. Phenotypic detection using liquid chromatography–tandem mass spectrometry (LC-MS/MS) can be used to detect tetracyclines degradation. However, this method cannot be used for high-throughput detection because of its complex sample pretreatment process [29]. Technological advancements and decreased sequencing costs allow WGS to replace a number of traditional microbiology laboratory methods, while the management and analysis of these large datasets still require specialized expertise and software tools [12]. In addition, compared with WGS method, MALDI-TOF/MS is also a rapid and reliable method for the identification of bacterial isolates [30].

To date, most studies have focused on the detection of new generation tetracyclines including TGC, ERA and OMA. Nevertheless, there are few studies concerning the detection of different tetracycline-resistant strains. While new-generation tetracyclines were minimally affected by the presence of tetracycline ribosomal protection or major efflux determinants in both Gram-positive and Gram-negative bacteria, early-generation tetracyclines (TC, OTC and DOX) have still risen in resistance by efflux or ribosomal protection [31,32]. Tetracyclines, including TC and OTC are frequently detected in natural waters, soils, and sediments that raise great concerns about ARG proliferation [33]. In this work, Tet(X)-producing strains can be specifically identified among the tetracycline-resistant strain. Firstly, the TGC was first used as an indicator to distinguish the Tet(X)-producing and non-producing bacteria. Then the non-Tet(X)-producing bacteria were subjected to co-incubation with OTC. The tetracycline-susceptible strains were found to degrade the OTC by generating intracellular ROS, yet the non-Tet(X)-producing tetracycline-resistant strains were barely capable to degrade OTC. Thus, the Tet(X)-producers, non-Tet(X) producing tetracycline-resistant bacteria and tetracycline-susceptible bacteria can be rapidly profiled by consecutive usage of TGC and OTC in this method. This is the first demonstration of the use of MALDI-TOF MS to distinguish Tet(X)-producers, non-Tet(X)-producing tetracycline-resistant strains, and tetracycline-susceptible strains.

In our developmental study we examined strains that possessed efflux pump mechanisms [*tet*(A), *tet*(B), *tet*(D), *tet*(G)], ribosomal protection mechanisms [tet(M)], and enzymatic modification mechanisms [*tet*(X3), *tet*(X4), *tet*(X2)-*tet*(X6), *tet*(X3)-*tet*(X6)] for tetracycline resistance, there are additional tetracycline ARGs possessed by Gram-negative bacteria that have not been tested. In theory, the MALDI^Tet(X)-plus^ test can be extended to examine additional isotypes. For instance, TmexCD1-toprJ1 is a novel plasmid-mediated multidrug resistance RND family efflux pump gene that confers resistance to tetracyclines including TGC and ERA, and possessed reduced susceptibility to many other clinically important antimicrobial agents used to treat Enterobacteriaceae infections [34]. The *E. coli* TMexCD1-TOprJ1 we examined using the MALDI^Tet(X)-plus^ test was classified as a non-Tet(X)-producing tetracycline-resistant strains as expected. The further evaluation of the MALDI^Tet(X)-plus^ test can be extended for the direct detection of additional isotypes. The MALDI^Tet(X)-plus^ data and method are potent to construct a supplementary library for fast phenotyping of antibiotic-resistant bacteria. And this method should be extended for the direct detection of these organisms and their metabolites in blood, urine samples as well as various types of food products [35,36]. In this regard, the current method is greatly potential to be implemented into those generally-applicable database, thereafter better fingerprinting the bacteria of interest.

An interesting finding is, more OTC degraded by the tetracycline-susceptible bacteria than that by non-Tet(X)-producing tetracycline-resistant bacteria. In the current study, the OTC metabolites was detected after co-incubation of OTC and the tetracycline-susceptible strains, suggesting the degradation of OTC even occurred in the strains absent of modes of action to catalyze OTC. Notably, the ROS accumulation in bacterial cells has been generally observed during the TCs exposure in several studies [37,38]. Moreover, the degradation of OTC by ROS, for example, the photo-Fenton process, were also intensively reported in previous publications and showed the same peaks of product as our work [39,40]. Therefore, we hypothesized that the ROS produced by stressed bacteria were responsible for the OTC degradation promoted by the non-Tet(X)-producers. It explained the rationale why the tetracycline-susceptible bacteria showed higher OTC-degradation than the non-Tet(X) producing tetracycline-resistant bacteria, in which less ROS was supposed to be generated due to the efflux pump or other mechanisms.

## Conclusions

In this work, we have designed the MALDI^Tet(X)-plus^ test method using MALDI-TOF MS for phenotypic detection to distinguish Tet(X)-producers from non-Tet(X)-producing tetracycline-resistant Gram-negative bacteria within 3 h and we found ROS played an important role in degrading OTC by tetracycline-susceptible strains. Its high-throughput feature allows 384 samples to be simultaneously examined. The calculation of the MS ratios M/(M + T) and M/(M + O) in three independent experiments is a simple, rapid, effective, high-throughput and low-cost method with excellent sensitivity and specificity.

## Supplementary Material

Supplemental Material

## Data Availability

The authors confirm that the data supporting the findings of this study are available within the article and its supplementary materials.
