# Supplementary figures and images for: MALDI-TOF MS for rapid detection and differentiation between Tet(X)-producers and non-Tet(X)-producing tetracycline-resistant Gram-negative bacteria
Source: Virulence. 2021 Dec 24;13(1):77–88. doi: 10.1080/21505594.2021.2018768 (PMC9794003; doi:10.1080/21505594.2021.2018768)

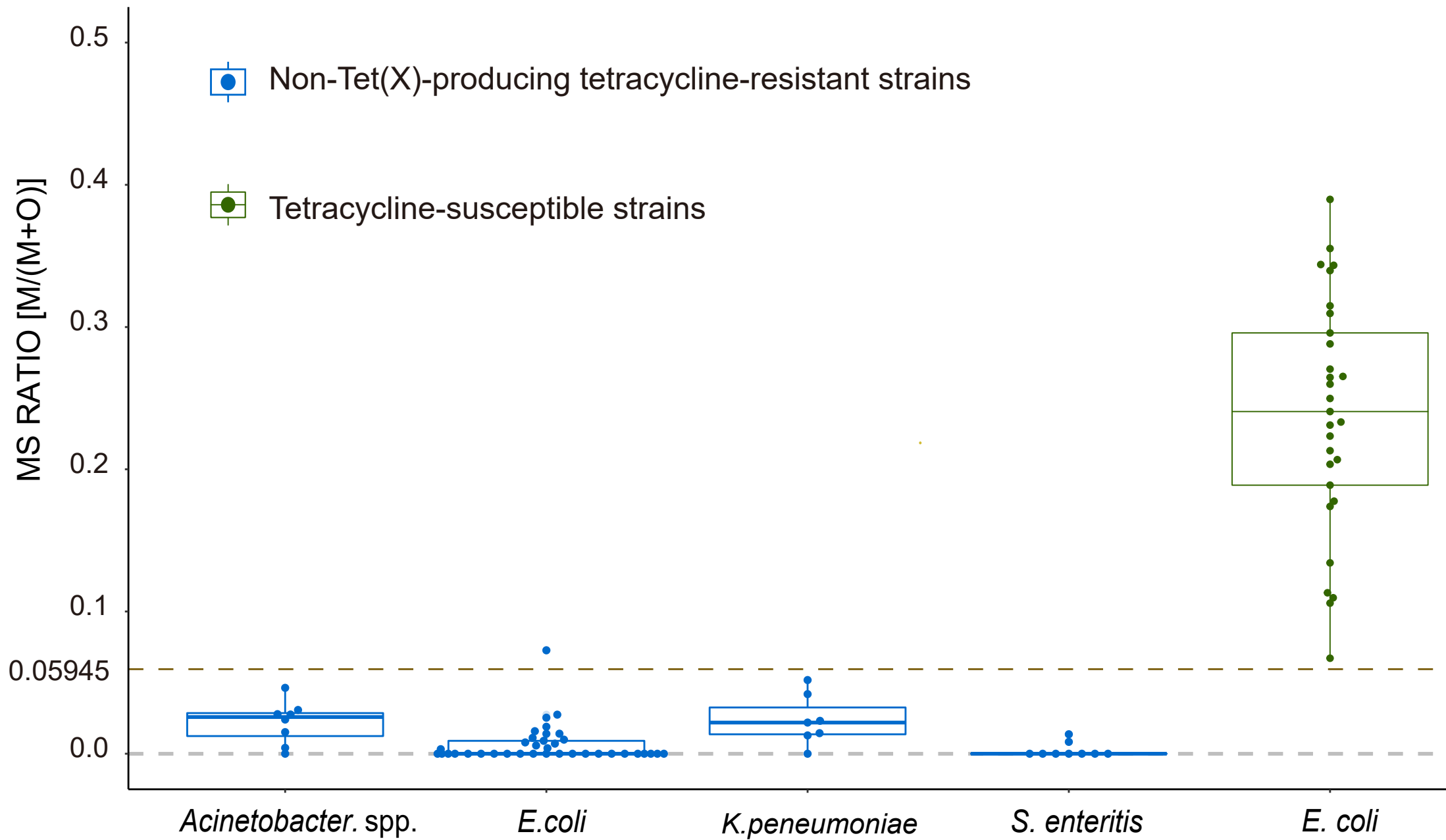

Supplement: Supplemental Material [file KVIR_A_2018768_SM6130.zip › supplementary/Supplementary_Figure_S1.pdf]

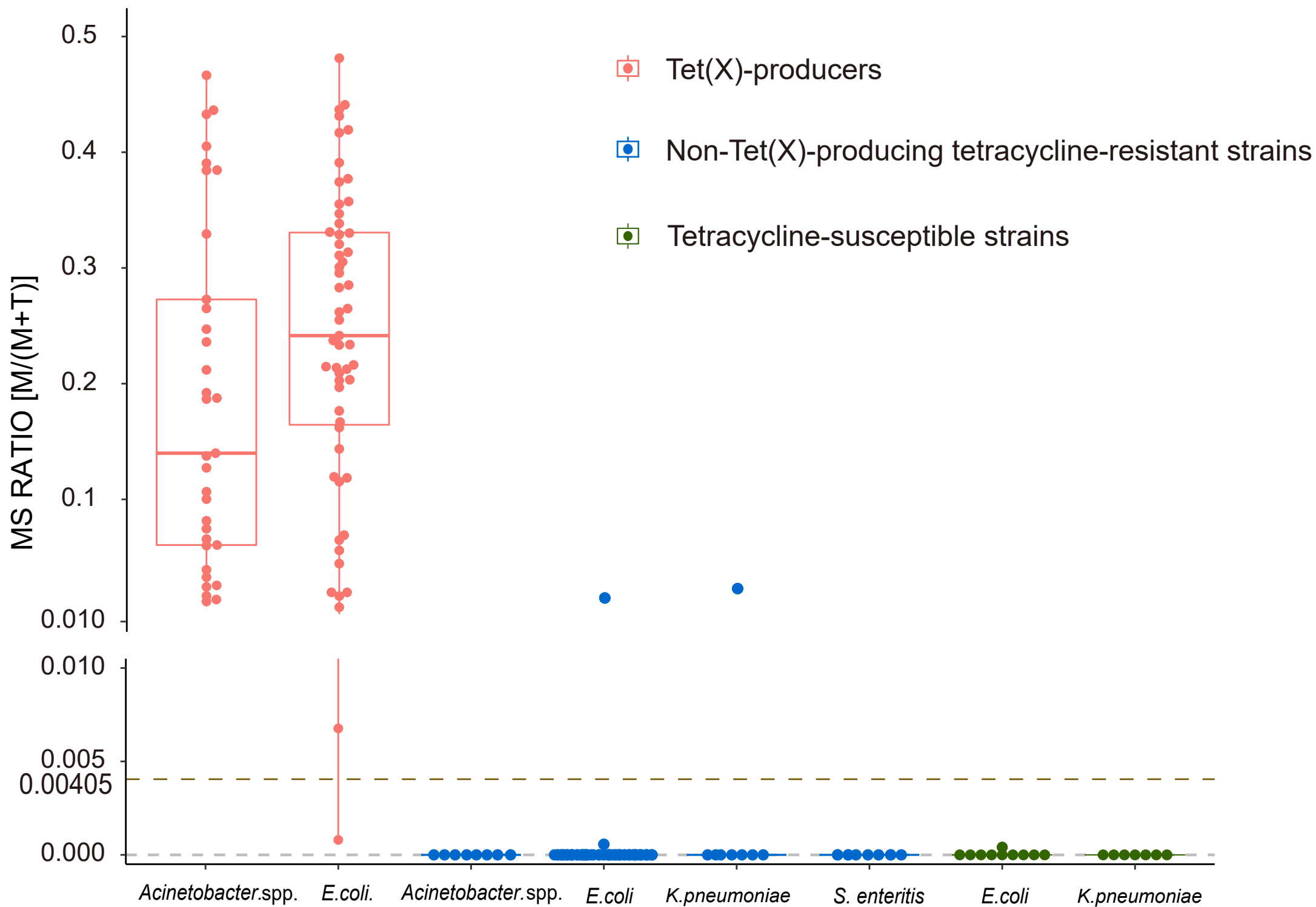

Supplement: Supplemental Material [file KVIR_A_2018768_SM6130.zip › supplementary/Supplementary_Figure_S2.pdf]

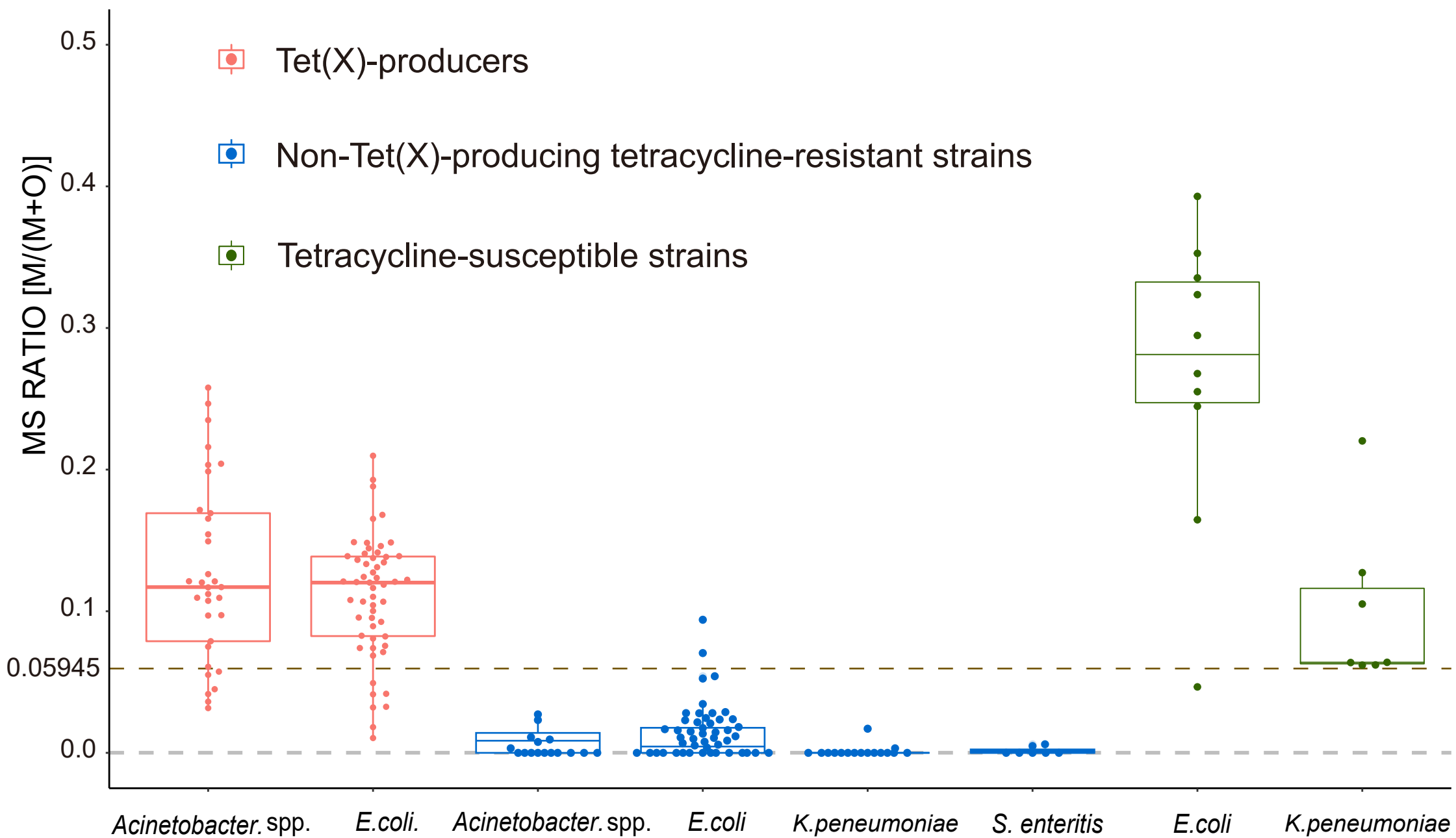

Supplement: Supplemental Material [file KVIR_A_2018768_SM6130.zip › supplementary/Supplementary_Figure_S3.pdf]
